# Functional Analysis of Feedback Inhibition-Insensitive Variants of *N*-Acetyl Glutamate Kinase Found in Sake Yeast Mutants with Ornithine Overproduction

**DOI:** 10.1128/spectrum.00822-22

**Published:** 2022-05-11

**Authors:** Masataka Ohashi, Shota Isogai, Hiroshi Takagi

**Affiliations:** a Nara Prefecture Institute of Industrial Development, Nara, Nara, Japan; b Division of Biological Science, Graduate School of Science and Technology, Nara Institute of Science and Technology, Ikoma, Nara, Japan; University of Minnesota

**Keywords:** *Saccharomyces cerevisiae*, sake yeast, ornithine, *N*-acetyl glutamate kinase, allosteric regulation, feedback inhibition

## Abstract

In the yeast Saccharomyces cerevisiae, *N*-acetyl glutamate kinase (NAGK), which catalyzes the phosphorylation of *N*-acetyl glutamate to form *N*-acetyl glutamyl-5-phosphate, is one of the rate-limiting enzymes in the ornithine and arginine biosynthetic pathways. NAGK activity is strictly regulated via feedback inhibition by the end product, arginine. We previously reported that the Thr340Ile variant of NAGK was insensitive to arginine feedback inhibition and that the interaction between Lys336 and Thr340 in NAGK may be important for arginine recognition. In the present study, we demonstrated that amino acid changes of Thr340 to Ala, Leu, Arg, Glu, Ile, and Asn removed arginine feedback inhibition, although the Thr340Ser variant was subject to the feedback inhibition. Therefore, these results indicate that the arginine-binding cavity formed via the interaction between the carbonyl group in the main chain of Lys336 and the hydroxyl group in the side chain of the residue at position 340 is critical for arginine recognition of NAGK. In addition, we newly identified two mutations in the *ARG5,6* gene encoding the Cys119Tyr or Val267Ala variant of NAGK of sake yeast mutants with intracellular ornithine accumulation. Although it is unlikely that Cys119 and Val267 are directly involved in arginine recognition, we found here that two variants of NAGK were insensitive to arginine feedback inhibition and contributed to high-level production of ornithine. Structural analysis of NAGK suggests that these two amino acid substitutions influence the sensitivity to Arg feedback inhibition through alterations in local conformation around each residue.

**IMPORTANCE** Ornithine has a number of physiological benefits in humans. Thus, an Orn-rich alcoholic beverage is expected to relieve feelings of fatigue after drinking. In the yeast Saccharomyces cerevisiae, *N*-acetyl glutamate kinase (NAGK) encoded by the *ARG5,6* gene catalyzes the second step in ornithine and arginine biosynthesis, and its activity is subjected to feedback inhibition by arginine. Here, we revealed a role of key residues in the formation of the arginine-binding cavity which is critical for arginine recognition of NAGK. In addition, we analyzed novel arginine feedback inhibition-insensitive variants of NAGK in sake yeast mutants with ornithine overproduction and proposed that the amino acid substitutions in the NAGK variants destabilize the arginine-binding cavity, leading to the lower sensitivity to arginine feedback inhibition of NAGK activity. These findings provide new insight into the allosteric regulation of NAGK activity and will help to construct superior industrial yeast strains for high-level production of ornithine.

## INTRODUCTION

Ornithine (Orn), a nonproteinogenic amino acid, functions in the urea cycle, which detoxifies ammonia generated by the degradation of amino acids in protein to urea. Various organisms and foods contain large amounts of Orn (1–3). In humans, Orn has been reported to have a number of physiological benefits, such as reducing the plasma ammonia level after bicycle exercise (4), improving various negative feelings (4, 5), enhancing sleep quality (6, 7), and increasing growth hormone levels (4, 8).

**FIG 1:**
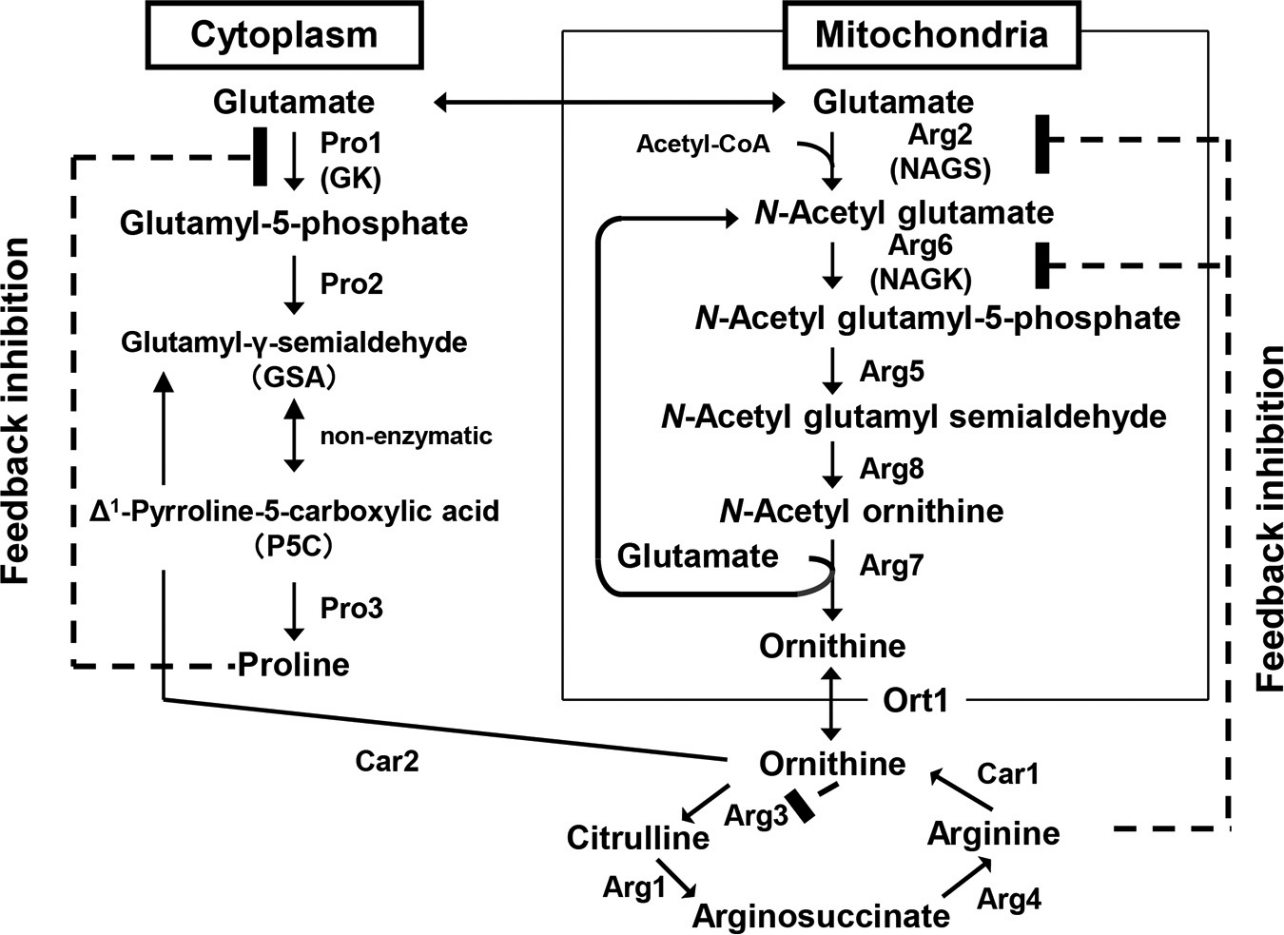
Metabolic pathways of proline (Pro), ornithine (Orn), and arginine (Arg) in S. cerevisiae. Protein names: Pro1, γ-glutamyl kinase (GK); Pro2, γ-glutamyl phosphate reductase; Pro3, P5C reductase; Arg2, *N*-acetyl glutamate synthase (NAGS); Arg6, *N*-acetyl glutamate kinase (NAGK); Arg5, *N*-acetyl glutamyl-5-phospate reductase; Arg8, *N*-acetyl Orn aminotransferase; Arg7, *N*-acetyl Orn acetyltransferase; Ort1, Orn transporter; Arg3, Orn carbamoyltransferase; Arg1, arginosuccinate synthetase; Arg4, arginosuccinate lyase; Car1, arginase; Car2, Orn aminotransferase. Acetyl-CoA, acetyl coenzyme A.

The budding yeast Saccharomyces cerevisiae synthesizes Orn from glutamate (Glu) via intermediates such as *N*-acetyl l-glutamate (NAG) and *N*-acetyl glutamyl-5-phosphate, *N*-acetyl glutamyl-γ-semialdehyde, and *N*-acetyl Orn in mitochondria (Fig. 1). The *ARG5*,*6* gene encodes a polyprotein precursor that is likely cleaved into two enzymes, one being *N*-acetyl glutamate kinase (NAGK, Arg6) and the other being *N*-acetyl glutamyl-5-phosphate reductase (Arg5), between residues 510 and 540 (9). NAGK is one of the rate-limiting enzymes of Orn and arginine (Arg) biosynthesis. This enzyme catalyzes the conversion of NAG into *N*-acetyl glutamyl-5-phosphate, and its activity is subject to feedback inhibition by Arg (10). *N*-Acetyl glutamate synthase (NAGS), which is encoded by the *ARG2* gene, is another rate-limiting step of Arg biosynthesis. NAGS transfers the acetyl group to Glu to produce NAG, and the protein or expression level of this enzyme is tightly regulated due to the Arg feedback inhibition (10) (Fig. 1). NAG can be regenerated by transferring the acetyl group from *N*-acetyl Orn to Glu with the production of Orn in S. cerevisiae (10) even if the synthesis of NAG by NAGS is limited by Arg (Fig. 1). Therefore, expressing NAGK variants that are desensitized to feedback inhibition is effective for overproduction of Orn, rather than expressing the feedback inhibition-insensitive NAGS variants. Alternatively, S. cerevisiae also synthesizes proline (Pro) from Glu. γ-Glutamyl kinase (GK) encoded by the *PRO1* gene is the rate-limiting enzyme of Pro biosynthesis from Glu, and its activity is strictly regulated by Pro feedback inhibition (11). Moreover, Pro can be biosynthesized from Orn via glutamyl-γ-semialdehyde by Orn transaminase activity (12) (Fig. 1).

Using traditional chemical mutagenesis, we recently isolated two Orn- and Pro-accumulating mutants (strains A902-4 and A902-6) that were resistant to the Pro toxic analogue azetidine-2-carboxylate (AZC) derived from a diploid sake yeast strain of S. cerevisiae (13). In addition, strain A902-4 had a homoallelic mutation in the *ARG5*,*6* gene encoding the feedback inhibition-insensitive variant of NAGK (Thr340Ile). Furthermore, our structural analysis of the Thr340Ile variant of NAGK predicted that the Arg-binding cavity consists of Lys265, Ser285, Lys336, Glu337, Thr340, and Gly345 and that this cavity may be stabilized through the interaction between the hydroxyl group in the side chain of Thr340 and the carbonyl group in the main chain of Lys336 (13).

In this study, to examine the importance of the amino acid residue at position 340, we analyzed the effects of amino acid substitution of Thr340 on NAGK activity and structure. In addition, we newly isolated another Orn-overproducing mutant (strain A902-8-3) that was resistant to AZC by conventional mutagenesis. Strains A902-6 and A902-8-3 each had a novel mutation in the *ARG5*,*6* gene encoding the Cys119Tyr and Val267Ala variants of NAGK, respectively, leading to an increase in intracellular Orn. Several genetically modified microorganisms, such as Corynebacterium crenatum (14) and S. cerevisiae (15), have been developed to overproduce Orn, using metabolic engineering or modular pathway rewiring. However, these strains have not been directly applied in food production because the food industry and customers still have not accepted genetically modified microorganisms. In contrast, the Orn-overproducing mutants (strains A902-4, A902-6, and A902-8-3) could be readily applied to sake brewing because they are not genetically modified.

## RESULTS AND DISCUSSION

### Isolation of sake yeast strains with intracellular Orn accumulation from AZC-resistant mutants.

**FIG 2:**
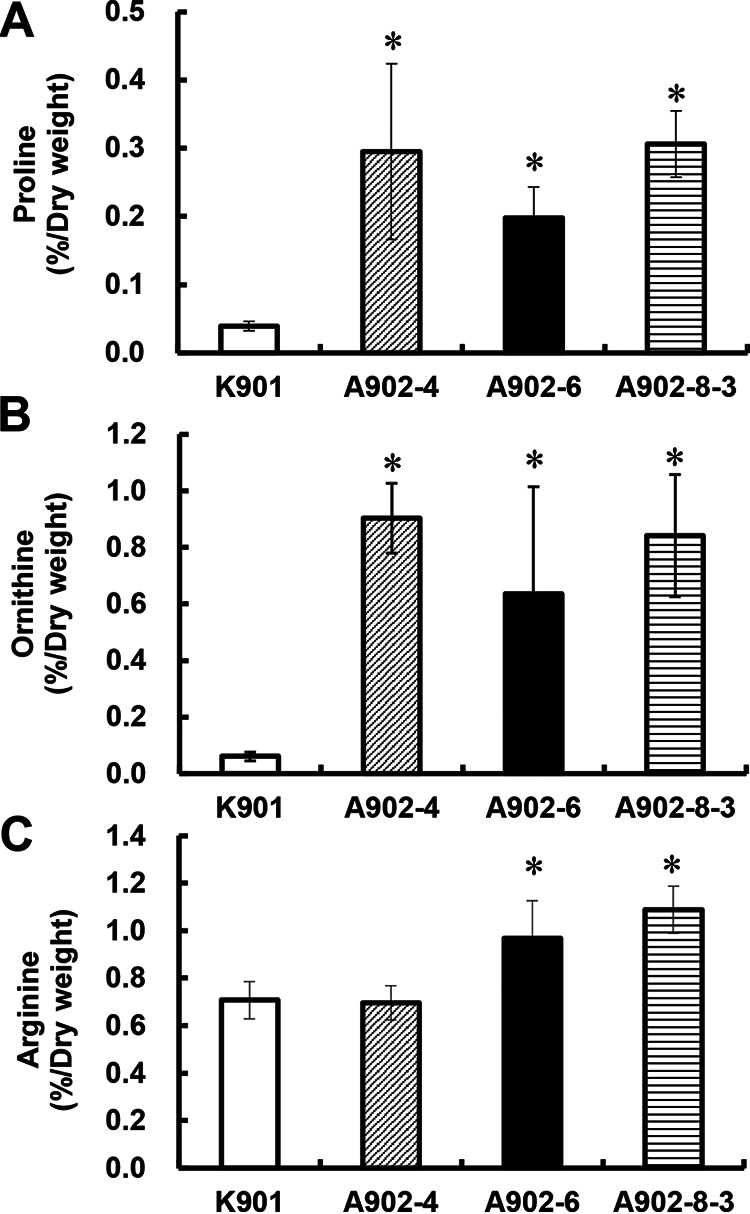
Intracellular Pro (A), Orn (B), and Arg (C) contents in sake yeast strains. Yeast cells were cultured in SD liquid medium at 30°C for 48 h. The values are means and standard deviations for results from three independent experiments. Asterisks indicate statistically significant differences between the parent strain K901 and the AZC-resistant mutant strains A902-4, A902-6, and A902-8-3 (Student’s *t* test, *P < *0.05).

In the present study, using traditional chemical mutagenesis, we isolated a new Pro- and Orn-accumulating strain, A902-8-3, from AZC-resistant mutants of diploid sake yeast strain K901 in addition to previously isolated strains A902-4 and A902-6 (13). As shown in Fig. 2A, the intracellular Pro contents in A902-4, A902-6, and A902-8-3 were 7.6-, 5.1-, and 7.8-fold higher, respectively, than that in the parent strain K901. Interestingly, these three strains accumulated Orn in the cells 14.8-, 10.4-, and 13.8-fold more than K901 (Fig. 2B). However, levels of intracellular Arg, which is biosynthesized from Orn, in these strains were not remarkably higher than in strain K901 (Fig. 2C).

### Identification of mutations in the *ARG5*,*6* gene of sake yeast strains with intracellular Orn accumulation.

Strain A902-4 was reported to carry a mutation in the *ARG5*,*6* gene encoding the Thr340Ile variant of NAGK. The NAGK activity of this variant was insensitive to feedback inhibition by Arg, leading to overproduction of Orn (13). Therefore, we assumed that two other strains, A902-6 and A902-8-3, also had one or more mutations in the *ARG5*,*6* gene. We therefore analyzed the nucleotide sequence of *ARG5*,*6* in each of these strains using direct PCR DNA sequencing (see Fig. S1 in the supplemental material). As expected, the *ARG5*,*6* gene sequence in A902-6 contained a homozygous mutation, which was a single base change at position 356, leading to one amino acid change of Cys to Tyr at position 119. On the other hand, A902-8-3 had a heterozygous mutation, i.e., a single base change at position 800, which led to one amino acid change of Val to Ala at position 267. In contrast, strains A902-6 and A902-8-3 harbored no other mutations in the genes encoding the rate-limiting steps for Orn (*ARG2*) and Pro (*PRO1*) biosynthesis (Fig. 1). Thus, we hypothesized that mutations identified in the *ARG5*,*6* gene of these two strains contribute to an increase in intracellular Pro and Orn levels.

### Effects of the *ARG5*,*6* mutations on intracellular amino acid contents.

To analyze the *ARG5*,*6* gene mutation found in strains A902-6 and A902-8-3, we constructed expression plasmids for the wild-type (WT) and mutated *ARG5*,*6* genes (*ARG5*,*6*^C119Y^ and *ARG5*,*6*^V267A^). In addition, the importance of Thr340 in the mechanism underlying the feedback inhibition of NAGK was still unknown. Therefore, we also designed a series of mutated *ARG5*,*6* genes (*ARG5*,*6*^T340A^, *ARG5*,*6*^T340L^, *ARG5*,*6*^T340R^, *ARG5*,*6*^T340E^, *ARG5*,*6*^T340N^, *ARG5*,*6*^T340I^, and *ARG5*,*6*^T340S^) that replaced Thr340 with various amino acids (Ala, Leu, Arg, Glu, Asn, Ile, and Ser) that have a side chain with a similar or different size, charge, and polarity.

**FIG 3:**
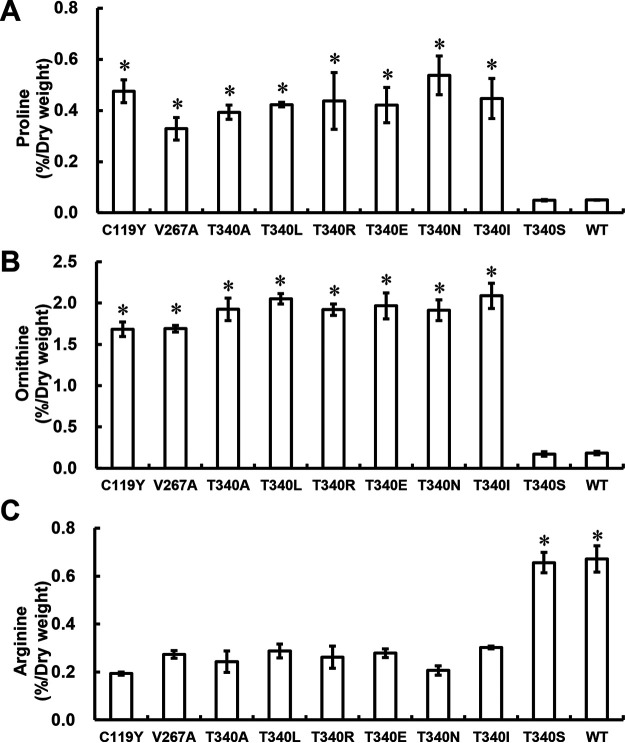
Intracellular Pro (A), Orn (B), and Arg (C) contents in laboratory yeast strains. BY4741 *arg5*,*6*Δ cells expressing the wild-type (WT) and mutated *ARG5*,*6* (C119V, V267A, T340A, T340L, T340R, T340E, T340N, T340I, and T340S) genes were cultured in SD+Leu+His+Met liquid medium at 30°C for 48 h. The values are means and standard deviations for results from three independent experiments. Asterisks indicate statistically significant differences between BY4741 *arg5*,*6*Δ cells expressing the WT and mutated *ARG5*,*6* genes (Student’s *t* test, *P < *0.05).

We next determined the intracellular amino acid contents in strain BY4741 *arg5*,*6*Δ expressing the wild-type and mutated *ARG5*,*6* genes (Fig. 3). Yeast cells expressing the mutated *ARG5*,*6* genes except for *ARG5*,*6*^T340S^ showed from 6.6- to 10.7-fold increases in Pro content compared to the wild-type *ARG5*,*6* gene (Fig. 3A). In addition, the expression of the mutated *ARG5*,*6* genes except for *ARG5*,*6*^T340S^ increased the intracellular Orn content markedly, in the range of 9.2- to 11.5-fold compared with that of BY4741 *arg5*,*6*Δ cells harboring the wild-type *ARG5*,*6* gene (Fig. 3B). The Pro and Orn levels in yeast cells expressing the *ARG5*,*6*^T340S^ gene were almost the same as those of cells expressing the wild-type *ARG5*,*6* gene (Fig. 3A and B). On the other hand, the Arg content in yeast cells expressing the mutated *ARG5*,*6* genes except for *ARG5*,*6*^T340S^ was significantly lower than that in cells expressing the wild-type *ARG5*,*6* gene (Fig. 3C). S. cerevisiae cells synthesize Arg from Orn via intermediates such as citrulline and arginosuccinate via the enzymatic reactions catalyzed by Arg3, Arg1, and Arg4 (Fig. 1). Among them, Orn carbamoyltransferase, encoded by the *ARG3* gene, catalyzes the conversion of Orn into citrulline, and excess Orn inhibits its enzyme activity (16). Therefore, this inhibition by excess Orn may result in a decrease in Arg level in cells expressing the mutated *ARG5*,*6* genes except for *ARG5*,*6*^T340S^. However, intracellular Arg levels in Orn-accumulating mutants were similar to (A902-4) or higher than (A902-6 and A902-8-3) that in the parent strain, K901 (Fig. 2C), suggesting that these mutants might cancel the excess Orn-mediated inhibition of Orn carbamoyltransferase activity. Besides the inhibition of the enzymatic activity of Arg3 by Orn, transcription of the *ARG1* and *ARG3* genes is repressed by Arg (17). One possibility for differences in intracellular Arg levels among the Orn-accumulating mutants thus is that such Arg-mediated transcriptional repression of the *ARG1* and *ARG3* genes may be abolished in strains A902-6 and A902-8-3, enabling the expression of these genes even in the presence of Arg. Further studies are needed to clarify the underlying molecular mechanism.

These results in Fig. 3A and B indicate that amino acid changes from Cys to Tyr at position 119, from Val to Ala at position 267, and from Thr to Ala, Leu, Arg, Glu, Asn, and Ile at position 340 in NAGK confer Pro and Orn accumulation on yeast cells.

### Effect of amino acid substitutions on NAGK activity.

We previously showed that the mutated *ARG5*,*6*^T340I^ gene encoding the Thr340Ile variant of NAGK elevated the intracellular Orn level due to the desensitization of feedback inhibition of NAGK activity (13). We therefore hypothesized that the amino acid substitutions at positions 119, 267, and 340 led to the desensitization of feedback inhibition of NAGK by Arg. To prove this hypothesis, we expressed the wild-type and mutated *ARG5*,*6* genes in Escherichia coli BL21(DE3) cells, purified the recombinant wild type (WT) and variants (C119Y, V267A, T340A, T340L, T340R, T340E, T340N, T340I, and T340S) of NAGK tagged with 6×His at the amino terminus from E. coli cell extracts (Fig. S2), and measured enzyme activities of NAGKs under conditions without or with Arg. As shown in Table 1, the specific activities of all nine NAGK variants, which ranged from 0.96 to 1.39 U/mg, were slightly lower than that of WT (1.57 U/mg) in the absence of Arg.

**TABLE 1:** Specific activities and arginine inhibition kinetics

NAGK	Sp act^*a*^ (U/mg)	IC_50_^Arg^^*b*^ (mM)
C119Y	0.96 ± 0.01	0.78 ± 0.05
V267A	1.39 ± 0.01	2.83 ± 0.16
T340A	1.27 ± 0.09	2.25 ± 0.20
T340L	1.35 ± 0.06	10.46 ± 0.80
T340R	1.16 ± 0.09	2.48 ± 0.67
T340E	0.98 ± 0.04	6.87 ± 1.00
T340N	1.20 ± 0.04	23.98 ± 5.36
T340I	1.27 ± 0.07	>100
T340S	1.25 ± 0.02	0.17 ± 0.12
WT	1.57 ± 0.03	0.09 ± 0.38

a Specific activity in the absence of arginine.

b The IC_50_^Arg^ value is an arginine concentration which gives 50% inhibition.

Judging from the results of Arg inhibition kinetics (Table 1), however, the half-maximal inhibitory concentration values of Arg (IC_50_^Arg^) of WT and T340S were less than 0.2 mM. In contrast, the newly identified variants (C119Y and V267A) exhibited higher IC_50_^Arg^ (0.78 mM and 2.83 mM, respectively) than that of WT, suggesting that Cys119 and Val267 are important for the regulation of NAGK activity. These results indicate that the novel NAGK variants (C119Y and V267A) were less sensitive to feedback inhibition by Arg than WT. Moreover, in the NAGK variants at position 340 (T340A, T340L, T340R, T340E, and T340N), each IC_50_^Arg^ (ranging from 0.78 mM to 23.98 mM) was higher than those of the WT and T340S enzymes. It should be noted that the IC_50_^Arg^ of the T340I variant (13) was the highest among all enzymes (>100 mM). We demonstrated that amino acid changes at position 340 (T340A, T340L, T340R, T340E, and T340N) resulted in less sensitivity to the Arg feedback inhibition of NAGK activity, leading to the accumulation of Orn in yeast cells.

Desensitization to the Arg feedback inhibition of NAGK led to overproduction of *N*-acetyl Orn and thereby enabled regeneration of NAG from Glu and *N*-acetyl Orn even though the synthesis of NAG is limited by the Arg feedback inhibition of NAGS, leading to Orn overproduction in strains A902-6 and A902-8-3 (Fig. 2B). In addition, strains A902-6 and A902-8-3 accumulated not only Orn but also Pro (Fig. 2A). Strains A902-4, A902-6, and A902-8-3 carry no mutations associated with Pro accumulation in the *PRO1* gene; thus, GK in these mutants is subject to feedback inhibition by Pro. These strains accumulated Pro probably due to biosynthesis of Pro from excess Orn (Fig. 1).

### Effects of amino acid substitutions on the NAGK structure.

Comparison of amino acid sequences among Arg-sensitive and -insensitive NAGKs demonstrates that Thr or Ser at position 340 is highly conserved among Arg-sensitive NAGKs including S. cerevisiae NAGK (18–20), whereas the corresponding residues contain no hydroxyl group in the side chain, such as Asn or Val, among Arg-insensitive NAGKs (21, 22) (Fig. S3). In addition, our results showed that NAGKs with the hydroxyl group in the side chain at position 340 (e.g., Thr and Ser) were sensitive to Arg feedback inhibition, suggesting the importance of the interaction between the hydroxyl group in the side chain of the residue at position 340 and the carbonyl group in the main chain of Lys336 for Arg recognition as described in our previous study (13).

**FIG 4:**
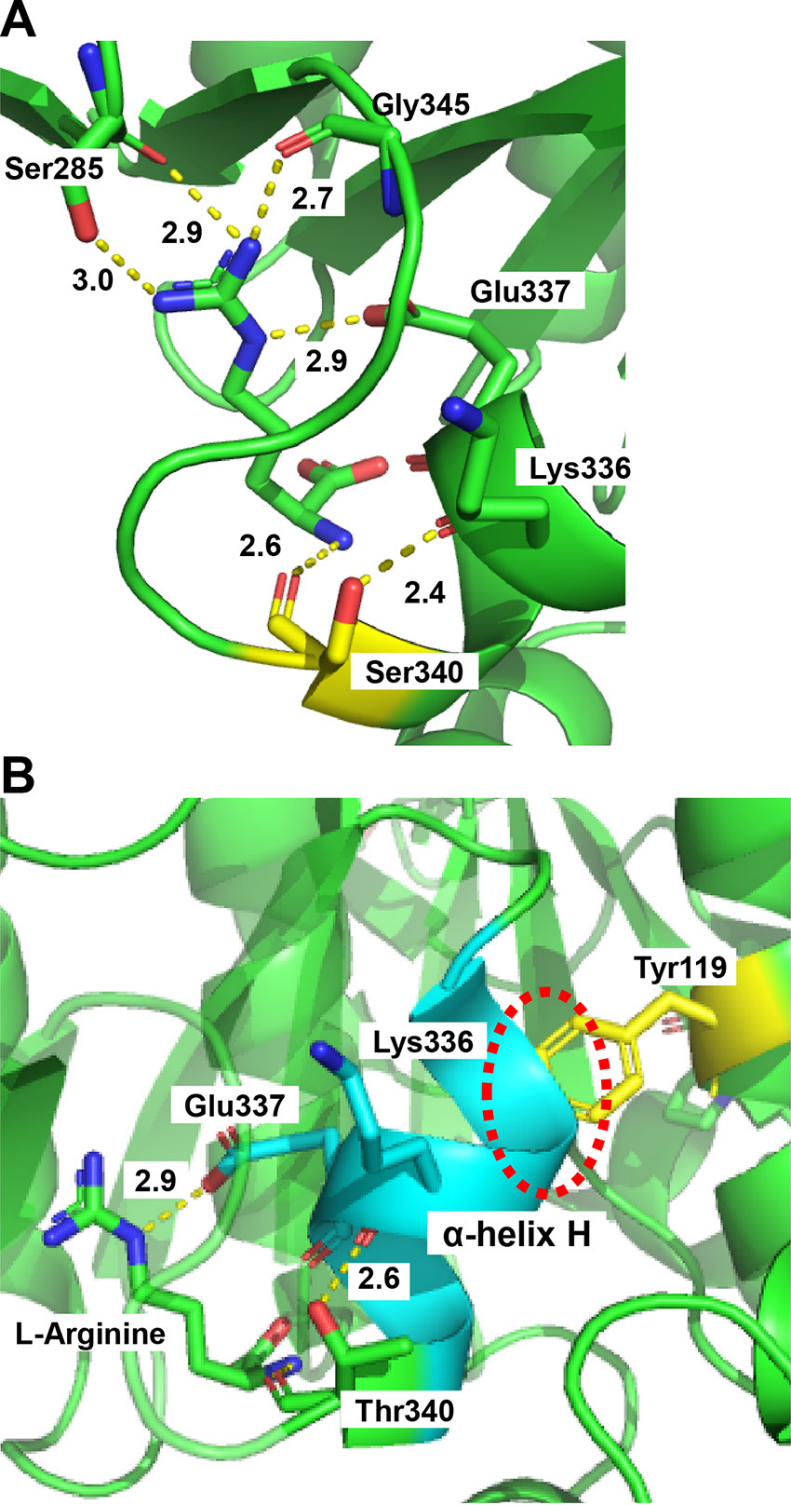
Predicted structure of Arg-binding site in S. cerevisiae NAGK variants. The Arg-binding sites of two NAGK variants, Thr340Ser (A) and Cys119Tyr (B), predicted from the structure of the S. cerevisiae NAGK (PDB ID, 3ZZH), are shown by the cartoon model. Arg, an inhibitor bound to NAGK, and the residues which interact with the inhibitor Arg are shown by the stick model. The replaced residues Ser340 (A) and Tyr119 (B) are shown in yellow. The expected hydrogen bonds are shown in yellow dots with their distance in angstroms. The αH-helix (Asp333-Phe339) including Lys336 is shown in cyan (B). A steric constraint is likely to arise from the αH-helix and the phenyl group of Tyr119 as shown by the dotted circle.

To confirm this hypothesis, we next predicted the structures of the Arg-binding sites of NAGK variants in which Thr340 was replaced with Ala, Leu, Arg, Glu, Asn, Ser, or Ile using the crystal structure of S. cerevisiae NAGK (PDB identifier [ID], 3ZZH) as a template. The results showed that replacement of Thr340 with Ser can still form the hydrogen bond with Lys336 as well as the WT enzyme because the hydroxyl group in the side chain of Ser340 is located near the carbonyl group in the main chain of Lys336 (2.4 Å) (Fig. 4A), resulting in sensitivity to feedback inhibition by Arg (Table 1). In contrast, the amino acid changes of Thr340 to Ala, Leu, Arg, Glu, Asn, and Ile cause disruption of the interaction of Lys336 with each of these amino acid residues as follows. The substitutions by Ala, Leu, and Ile cannot form the hydrogen bond with Lys336 due to the lack of a hydroxyl group in the side chain of these amino acids, whereas those by Arg, Glu, and Asn are also unlikely to form based on the predicted distances from the carbonyl group in the main chain of Lys336 to the imino group (4.4 Å), the carboxyl group (5.9 Å), and the amino group (5.0 Å) in the side chain of Arg340, Glu340, and Asn340, respectively. These results indicate that the interaction between the hydroxyl group in the side chain of the residue at position 340 and the carbonyl group in the main chain of Lys336 is important for the stabilization of the structure of the Arg-binding site in the S. cerevisiae NAGK. The IC_50_^Arg^ of uncharged and bulky amino acids (Ile, Asn, and Leu) in the side chain was higher than that of the acidic (Glu), the basic (Arg), or the small (Ala) amino acid in the side chain (Table 1). These findings can result from the differences in the effects of neutral and bulky amino acids and charged or small amino acids on the Arg-binding cavity. Particularly, Ile340 might completely disrupt the Arg-binding cavity, judging from the high IC_50_^Arg^.

Moreover, we predicted the structure of the Arg-binding site in the C119Y and V267A variant. It is unlikely that Cys119 and Val267 are directly involved in Arg recognition. However, the replacement of Cys119 with tyrosine may cause the steric constraint between the phenolic group of Tyr119 and the αH-helix (Asp333-Phe339) including Lys336 and Glu337 involved in the interaction with Arg (Fig. 4B). The steric constraint caused by Cys-to-Tyr alteration thus could affect the structure of the Arg-binding site in NAGK, leading to the lower sensitivity to Arg feedback inhibition of NAGK in the C119Y variant. On the other hand, the previous structural analysis of S. cerevisiae NAGK reported that the binding of inhibitor Arg caused a conformational change with an axis that crosses obliquely the central β sheet (β11-sheet) including Val267-Tyr268, resulting in transition from the closed-active form to the open-inactive form (Fig. S4A) (23). One possibility is that substitution for Val267 by Ala may influence a local structure around the β11-sheet because of the smaller side chain of Ala than that of Val (Fig. S4B and C), and the presumed conformational change around the β11-sheet may affect the transition from the close-active to the open-inactive form by Arg, resulting in the desensitization to the Arg feedback inhibition. However, further structural studies, such as molecular dynamics analysis, are needed to clarify the mechanism underlying replacements of Cys119 with Tyr and of Val267 with Ala.

### Conclusion.

In the present study, functional analysis of the Thr340 variants of NAGK revealed that the Arg-binding cavity formed through the interaction between Lys336 and Thr340 is critical for Arg recognition of NAGK. In addition, we newly analyzed diploid sake yeast mutants A906-2 and A902-8-3, which overproduced not only Pro but also Orn. These two strains possessed novel mutations in the *ARG5*,*6* genes encoding the Cys119Tyr and Val267Ala variants of NAGK. These amino acid substitutions weakened the Arg feedback inhibition of NAGK, leading to high-level production of both Pro and Orn in yeast cells. Structural analysis suggests that the two amino acid replacements Cys119Tyr and Val267Ala desensitize Arg feedback inhibition through alterations in local conformation around each residue. These results will contribute to the development of superior industrial yeast strains for high-level production of Orn, which has a number of physiological benefits.

## MATERIALS AND METHODS

### Strains and media.

We used S. cerevisiae strain BY4741 (*MAT***a** *his3*Δ*1 leu2*Δ*0 met15*Δ*0 ura3*Δ*0*) derived from strain S288C and diploid strain Kyokai no. 901 (K901) in this study. Strain BY4741 *arg5*,*6*Δ (BY4741 *arg5*,*6*::*kan*MX6), which was provided by EUROSCARF (Oberursel, Germany), was used for the expression of the WT and mutated *ARG5*,*6* genes. The media used for growth of S. cerevisiae were a nutrient-rich YPD medium (10 g/L yeast extract, 20 g/L peptone, and 20 g/L glucose), a synthetic dextrose (SD) minimal medium (1.7 g/L nitrogen base without amino acid and ammonium sulfate [Difco Laboratories, Detroit, MI, USA], 5 g/L ammonium sulfate, and 20 g/L glucose), and SD medium containing 0.04% leucine, 0.008% histidine, and 0.008% methionine (SD+Leu+His+Met). AZC was added as a supplement at a concentration of 1 mg/mL for the screening of Orn-accumulating cells.

Escherichia coli strains DH5α [F^−^ λ^−^ ϕ80*lacZ*ΔM15 Δ(*lacZYA argF*) *U169 deoR recA1 endA1 hsdR17*(r_K_^−^ m_K_^+^) *supE44 thi-1 gyrA96*] and BL21(DE3) [F^−^ *ompT hsdS*(r_B_^−^ m_B_^−^) *gal dcm* λ(DE3) (*lacI lacUV5-T7 gene1 ind1 sam7 nin5*)] were used for construction of expression plasmids and for expression of the recombinant NAGKs, respectively. E. coli strains were cultured in Luria-Bertani (LB) medium (0.5% yeast extract, 1% tryptone, and 1% NaCl) containing 100 μg/mL ampicillin. If necessary, 2% agar was added to solidify the medium.

### Isolation of Orn-accumulating mutants.

Orn-accumulating mutants were isolated by conventional mutagenesis of the diploid sake strain K901 as described previously (13).

### Measurement of intracellular amino acid content.

We quantified intracellular amino acid content of S. cerevisiae cells with a UF-Amino Station (Shimadzu, Kyoto, Japan) as described previously (13).

### Construction of expression plasmids for the *ARG5*,*6* genes.

To construct plasmids for expressing the *ARG5*,*6* genes, the DNA fragment including 1,000 bp both upstream and then downstream of the open reading frame of the *ARG5*,*6* gene was amplified from the genomic DNA of S. cerevisiae BY4741 by high-fidelity PCR with primers ARG5,6FW (HindIII) and ARG5,6Rv (BamHI) (see Table S1 in the supplemental material) and was introduced into the HindIII-BamHI site of yeast centromere plasmid pRS416 by the In-Fusion HD cloning kit (TaKaRa Bio, Shiga, Japan) to construct pRS416-ARG5,6. In-Fusion products were then digested with *Dpn*I before introduction into E. coli DH5α cells. The point mutations were introduced into the *ARG5*,*6* gene on pRS416-ARG5,6 with mutagenic primers listed in Table S1, leading to the T340A, T340L, T340R, T340E, T340N, T340S, T340I, V267A, and C119Y substitutions on NAGK. PCR products were then digested with *Dpn*l before introduction into E. coli DH5α cells. Plasmids of pRS416 harboring the wild-type or mutated *ARG5*,*6* gene were further confirmed by DNA sequencing using the BigDye Terminator v3.1 cycle sequencing kit (Applied Biosystems, Waltham, MA, USA). Strain BY4741 *arg5*,*6*Δ was transformed with pRS416 harboring the wild-type or mutated *ARG5*,*6* gene by the lithium acetate method with slight modification (24). Yeast transformants were selected on SD+Leu+His+Met agar plates. To express and purify the recombinant NAGKs from E. coli, we constructed expression plasmids as described below. The *ARG5*,*6* gene encodes a polyprotein precursor with the mitochondrial targeting signal sequence (residues 1 to 57), the amino-terminal NAGK (residues 58 to 513), and the carboxyl-terminal *N*-acetyl glutamyl phosphate reductase (23). The polyprotein precursor is cleaved into the two enzymes in the mitochondria (9), and then deletion of the amino-terminal mitochondrial targeting signal sequence results in yeast mature NAGK by proteolytic processing. To produce NAGK, which lacks the mitochondrial targeting sequence, the DNA sequence encoding enzyme residues 58 to 513 was amplified from pRS416-ARG5,6 (T340A, T340L, T340R, T340R, T340E, T340N, T340S, T340I, V267A, and C119Y) by high-fidelity PCR using KOD Plus DNA polymerase with primers pQE-2 NdHi Fw (NdeI) and pQE-2 NdHi Rv (HindIII) (Table S1) and was cloned into the NdeI-HindIII site of pQE-2 (Qiagen, Hilden, Germany) by the In-Fusion HD cloning kit to construct pQE-2-NAGK-WT or pQE-2-NAGK-variants. In-Fusion products were then digested with *Dpn*l before introduction into DH5α cells. Plasmids pQE-2-NAGK-WT and pQE-2-NAGK-variants were further confirmed by DNA sequencing. E. coli strain BL21(DE3) was transformed with pQE-2-NAGK-WT or pQE-2-NAGK-variants.

### Expression and purification of recombinant NAGKs.

E. coli BL21(DE3) cells harboring pQE-2-NAGK-WT or pQE-2-NAGK-variants were cultivated in 5 mL of LB medium containing 100 μg/mL ampicillin and grown at 37°C to an optical density at 600 nm (OD_600_) of 0.6 to 0.8. Protein expression was induced by adding isopropyl-β-d-1-thiogalactopyranoside (IPTG) to a final concentration of 0.1 mM, and cells were cultured at 18°C for 18 h. Cells were harvested by being placed on ice for 5 min and centrifuged at 4°C for 10 min at 3,000 rpm. The cell pellet was washed with ice-cold sonication buffer (10 mM MgCl_2_, 0.25 M NaCl, 20 mM Tris-HCl [pH 7.5]), and the intact cells were stored at −80°C if they were not used immediately. For purification of recombinant NAGKs, cell pellets were suspended in ice-cold sonication buffer. The cell mixture was subjected to sonication (output = 7, 10 s/on ice 30 s for 1 cycle) by a US-150T ultrasonic generator (Nihonseiki, Tokyo, Japan), was subsequently centrifuged at 15,000 rpm for 20 min at 4°C, and then filtered with an 0.45-μm-diameter filter. Crude protein was loaded by gravity on Ni-Sepharose 6 Fast Flow resin (GE Healthcare, Chicago, IL, USA), equilibrated with sonication buffer. The resin was washed in sonication buffer with 20 mM imidazole and then with 70 mM imidazole-containing sonication buffer. Purified proteins were eluted by 500 mM imidazole-containing sonication buffer. Proteins were quantified using Bio-Rad protein assay (Hercules, CA, USA) and subjected to SDS-polyacrylamide gel electrophoresis (Fig. S2).

### Assay of NAGK activity.

NAGK activity was measured by an enzyme-coupled system with lactate dehydrogenase (LDH) and pyruvate kinase (PK) as previously described (17). Briefly, the production of ADP by NAGK was detected with the LDH-dependent oxidation of reduced NADH in the presence of phosphoenol pyruvate (PEP) and PK using a Synergy HTX microplate reader (Bio-Tek Instruments, Winooski, VT, USA). The NAGK reaction mixture contained 25 mM NAG, 5 mM ATP, 10 mM MgCl_2_, 1 mM PEP, 20 U PK, 30 U LDH enzymes from rabbit muscle (Sigma-Aldrich, St. Louis, MO, USA), 0.25 mM NADH, 100 mM HEPES (pH 7.5), and 5 μg of NAGK in a final volume of 300 μL. The NAGK reaction mixture was prewarmed at 30°C for 1 min without NAG, and then NAG was added to start the NAGK reaction. The rate of the decrease in absorption at 340 nm was measured consecutively. NAGK activity was calculated from the initial linear rate with the extinction coefficient (ε_340 nm_ = 6.22 mM^−1^ 1 cm^−1^) of NADH. One unit of activity was defined as the amount of enzyme required to produce 1 μmol of NAD^+^ per min. To determine the 50% inhibitory concentration (IC_50_) values of NAGK activity, NAGK activity was measured by adding Arg, and kinetic parameters of each enzyme were calculated with GraphPad Prism version 9 (GraphPad Software) using nonlinear regression analysis.

### Structural analysis.

To analyze the effect of amino acid substitution on NAGK, we predicted the structure of the Arg-binding site in the variant NAGKs using the crystal structure of NAGK bound to Arg from S. cerevisiae (PDB ID, 3ZZH) as the template structure as follows: Thr340 was replaced with Ala, Leu, Arg, Glu, Asn, Ser, and Ile, respectively, or Cys119 and Val267 were changed to Tyr119 and Ala267, respectively, using PyMOL (http://www.pymol.org). Figures of NAGK variant structures were drawn using PyMOL.

### Data availability.

The data underlying this article are available in the article.

## References

[1] Manzi P, Gambelli L, Marconi S, Vivanti V, Pizzoferrato L. 1999. Nutrients in edible mushrooms: an inter-species comparative study. Food Chem 65:477–482. doi:10.1016/S0308-8146(98)00212-X.

[2] Uchisawa H, Sato A, Ichita J, Matsue H, Ono T. 2004. Influence of low-temperature processing of the brackish-water bivalve, *Corbicula japonica*, on the ornithine content of its extract. Biosci Biotechnol Biochem 68:1228–1234. doi:10.1271/bbb.68.1228. 15215585

[3] Niro S, Succi M, Tremonte P, Sorrentino E, Coppola R, Panfili G, Fratianni A. 2017. Evolution of free amino acids during ripening of Caciocavallo cheeses made with different milks. J Dairy Sci 100:9521–9531. doi:10.3168/jds.2017-13308. 28941817

[4] Takeda K, Takemasa T. 2019. An overview of ornithine, arginine, and citrulline in exercise and sports nutrition, p 627–636. *In* Bagchi D, Nair S, Sen CK (ed), Nutrition and enhanced sports performance, 2nd ed. Academic Press, New York, NY.

[5] Kokubo T, Ikeshima E, Kirisako T, Miura Y, Horiuchi M, Tsuda A. 2013. A randomized, double-masked, placebo-controlled crossover trial on the effects of L-ornithine on salivary cortisol and feelings of fatigue of flushers the morning after alcohol consumption. Biopsychosoc Med 7:6. doi:10.1186/1751-0759-7-6. 23414576 PMC3583691

[6] Miyake M, Kirisako T, Kokubo T, Miura Y, Morishita K, Okamura H, Tsuda A. 2014. Randomised controlled trial of the effects of L-ornithine on stress markers and sleep quality in healthy workers. Nutr J 13:53. doi:10.1186/1475-2891-13-53. 24889392 PMC4055948

[7] Omori K, Kagami Y, Yokoyama C, Moriyama T, Matsumoto N, Masaki M, Nakamura H, Kamasaka H, Shiraishi K, Kometani T, Kuriki T, Huang Z, Urade Y. 2012. Promotion of non–rapid eye movement sleep in mice after oral administration of ornithine. Sleep Biol Rhythms 10:38–45. doi:10.1111/j.1479-8425.2011.00515.x.

[8] Demura S, Yamada T, Yamaji S, Komatsu M, Morishita K. 2010. The effect of L-ornithine hydrochloride ingestion on human growth hormone secretion after strength training. Adv Biosci Biotechnol 1:7–11. doi:10.4236/abb.2010.11002.

[9] Abadjieva A, Pauwels K, Hilven P, Crabeel M. 2001. A new yeast metabolon involving at least the two first enzymes of arginine biosynthesis: acetylglutamate synthase activity requires complex formation with acetylglutamate kinase. J Biol Chem 276:42869–42880. doi:10.1074/jbc.M103732200. 11553611

[10] Pauwels K, Abadjieva A, Hilven P, Stankiewicz A, Crabeel M. 2003. The *N*-acetylglutamate synthase/*N*-acetylglutamate kinase metabolon of *Saccharomyces cerevisiae* allows coordinated feedback regulation of the first two steps in arginine biosynthesis. Eur J Biochem 270:1014–1024. doi:10.1046/j.1432-1033.2003.03477.x. 12603335

[11] Sekine T, Kawaguchi A, Hamano Y, Takagi H. 2007. Desensitization of feedback inhibition of the *Saccharomyces cerevisiae* γ-glutamyl kinase enhances proline accumulation and freezing tolerance. Appl Environ Microbiol 73:4011–4019. doi:10.1128/AEM.00730-07. 17449694 PMC1932739

[12] Kaino T, Tasaka Y, Tatehashi Y, Takagi H. 2012. Functional analysis of the C-terminal region of γ-glutamyl kinase of *Saccharomyces cerevisiae*. Biosci Biotechnol Biochem 76:454–461. doi:10.1271/bbb.110682. 22451384

[13] Ohashi M, Nasuno R, Isogai S, Takagi H. 2020. High-level production of ornithine by expression of the feedback inhibition-insensitive *N*-acetyl glutamate kinase in the sake yeast *Saccharomyces cerevisiae*. Metab Eng 62:1–9. doi:10.1016/j.ymben.2020.08.005. 32805427

[14] Shu Q, Xu M, Li J, Yang T, Zhang X, Xu Z, Rao Z. 2018. Improved L-ornithine production in *Corynebacterium crenatum* by introducing an artificial linear transacetylation pathway. J Ind Microbiol Biotechnol 45:393–404. doi:10.1007/s10295-018-2037-1. 29728854

[15] Liu Q, Yu T, Campbell K, Nielsen J, Chen Y. 2018. Modular pathway rewiring of yeast for amino acid production. Methods Enzymol 608:417–439. doi:10.1016/bs.mie.2018.06.009. 30173772

[16] Messenguy F, Penninckx M, Wiame JM. 1971. Interaction between arginase and ornithine carbamoyltransferase in *Saccharomyces cerevisiae*. Eur J Biochem 22:277–286. doi:10.1111/j.1432-1033.1971.tb01542.x. 5116613

[17] Crabeel M, Lavalle R, Glansdorff N. 1990. Arginine-specific repression in *Saccharomyces cerevisiae*: kinetic data on *ARG1* and *ARG3* mRNA transcription and stability support a transcriptional control mechanism. Mol Cell Biol 10:1226–1233. doi:10.1128/mcb.10.3.1226-1233.1990. 2406564 PMC361004

[18] Beez S, Fokina O, Herrmann C, Forchhammer K. 2009. *N*-Acetyl-L-glutamate kinase (NAGK) from oxygenic phototrophs: PII signal transduction across domains of life reveals novel insights in NAGK control. J Mol Biol 389:748–758. doi:10.1016/j.jmb.2009.04.053. 19409905

[19] Ramon-Maiques S, Fernández-Murga ML, Gil-Ortiz F, Vagin A, Fita I, Rubio V. 2006. Structural bases of feed-back control of arginine biosynthesis, revealed by the structures of two hexameric *N*-acetylglutamate kinases, from *Thermotoga maritima* and *Pseudomonas aeruginosa*. J Mol Biol 356:695–713. doi:10.1016/j.jmb.2005.11.079. 16376937

[20] Huang Y, Zhang H, Tian H, Li C, Han S, Lin Y, Zheng S. 2015. Mutational analysis to identify the residues essential for the inhibition of *N*-acetyl glutamate kinase of *Corynebacterium glutamicum*. Appl Microbiol Biotechnol 99:7527–7537. doi:10.1007/s00253-015-6469-5. 25750030

[21] Cunin R, Glansdorff N, Piérard A, Stalon V. 1986. Biosynthesis and metabolism of arginine in bacteria. Microbiol Rev 50:314–352. doi:10.1128/mr.50.3.314-352.1986. 3534538 PMC373073

[22] Udaka S. 1966. Pathway-specific pattern of control of arginine biosynthesis in bacteria. J Bacteriol 91:617–621. doi:10.1128/jb.91.2.617-621.1966. 5883101 PMC314904

[23] de Cima S, Gil-Ortiz F, Crabeel M, Fita I, Rubio V. 2012. Insight on an arginine synthesis metabolon from the tetrameric structure of yeast acetylglutamate kinase. PLoS One 7:e34734. doi:10.1371/journal.pone.0034734. 22529931 PMC3329491

[24] Agatep R, Kirkpatrick RD, Parchaliuk DL, Woods RA, Gietz RD. 1998. Transformation of *Saccharomyces cerevisiae* by the lithium acetate/single-stranded carrier DNA/polyethylene glycol protocol. Tech Tips Online 3:133–137. doi:10.1016/S1366-2120(08)70121-1.

