# Supplementary material for: Functional Analysis of Feedback Inhibition-Insensitive Variants of N-Acetyl Glutamate Kinase Found in Sake Yeast Mutants with Ornithine Overproduction
Source: Microbiol Spectr. 2022 May 11;10(3):e00822-22. doi: 10.1128/spectrum.00822-22 (PMC9241915; doi:10.1128/spectrum.00822-22)
Supplement: SUPPLEMENTAL FILE 1 — Table S1; Fig. S1 to S4. Download spectrum.00822-22-s001.pdf, PDF file, 0.9 MB [file spectrum.00822-22-s001.pdf]

Supplemental Material

Functional Analysis of Feedback Inhibition-insensitive Variants of *N*-Acetyl Glutamate Kinase Found in Sake Yeast Mutants with Ornithine Overproduction

Masataka Ohashi[a], Shota Isogai[b], Hiroshi Takagi[b#]

[a] Nara Prefecture Institute of Industrial Development, 129-1 Kashiwagi-cho, Nara, Nara 630-8031, Japan

[b] Division of Biological Science, Graduate School of Science and Technology, Nara Institute of Science and Technology, 8916-5 Takayama, Ikoma, Nara 630-0192, Japan

Running title: Feedback inhibition-insensitive variants of *N*-acetyl glutamate kinase

#Address correspondence to Hiroshi Takagi, hiro@bs.naist.jp

This file includes TABLE S1, FIG S1-S4.

TABLE S1 Oligonucleotide primers used in this study

Primer	Sequence (5' → 3') [a]
ARG5,6 FW (*Hin* dIII)	CGG TAT CGA TAA GCT TAA GAA ACT TAT AAA AAG TAT CCG
ARG5,6 RV (*Bam* HI)	TAG AAC TAG TGG ATC CTC TTA TCG AAT TGG ACA GGT
Mutagenic primers	
ARG5,6 (T340A) FW	AAA GAA CTG TTC G*CT GAT TCT GGT GCG
ARG5,6 (T340A) RV	CGC ACC AGA ATC AGC* GAA CAG TTC TTT
ARG5,6 (T340L) FW	AAA GAA CTG TTC T*T*A* GAT TCT GGT GCG
ARG5,6 (T340L) RV	CGC ACC AGA ATC T*A*A GAA CAG TTC TTT
ARG5,6 (T340R) FW	AAA GAA CTG TTC AG*G* GAT TCT GGT GCG
ARG5,6 (T340R) RV	CGC ACC AGA ATC C*C*T GAA CAG TTC TTT
ARG5,6 (T340E) FW	AAA GAA CTG TTC G*A*A* GAT TCT GGT GCG
ARG5,6 (T340E) RV	CGC ACC AGA ATC T*T*C* GAA CAG TTC TTT
ARG5,6 (T340N) FW	AAA GAA CTG TTC AA*T GAT TCT GGT GCG
ARG5,6 (T340N) RV	CGC ACC AGA ATC AT*T GAA CAG TTC TTT
ARG5,6 (T340S) FW	AAA GAA CTG TTC T*CT GAT TCT GGT GCG
ARG5,6 (T340S) RV	CGC ACC AGA ATC AGA* GAA CAG TTC TTT
ARG5,6 (T340I) FW	AAG AAC TGT TCA T*TG ATT CTG GTG C
ARG5,6 (T340I) RV	GCA CCA GAA TCA A*TG AAC AGT TCT T
ARG5,6 (V267A) FW	CCT TTG AAG ATC GC*T TAC CTG AAT GAG
ARG5,6 (V267A) RV	CTC ATT CAG GTA AG*C GAT CTT CAA AGG
ARG5,6 (C119Y) FW	GAA CTC GCT TCC TA*C TTG GCA TTT TTG
ARG5,6 (C119Y) RV	CAA AAA TGC CAA GT*A GGA AGC GAG TTC
pQE-2 NdHi Fw (*Nde* I)	ATC ACC ATC ACC ATA TGG TTT CAT CTA CTA ACG GCT T
pQE-2 NdHi Rv (*Hin* dIII)	TCA GCT AAT TAA GCT TTC AAC TAC TTG CTG ATG AGT

[a] The single-underlined sequences indicate *Hin*dIII, *Bam*HI, *Nde*I, and *Hin*dIII restriction sites, respectively. The asterisks and the double-underlined sequences indicate the positions of the nucleotide mutation and the amino acid substitution, respectively.

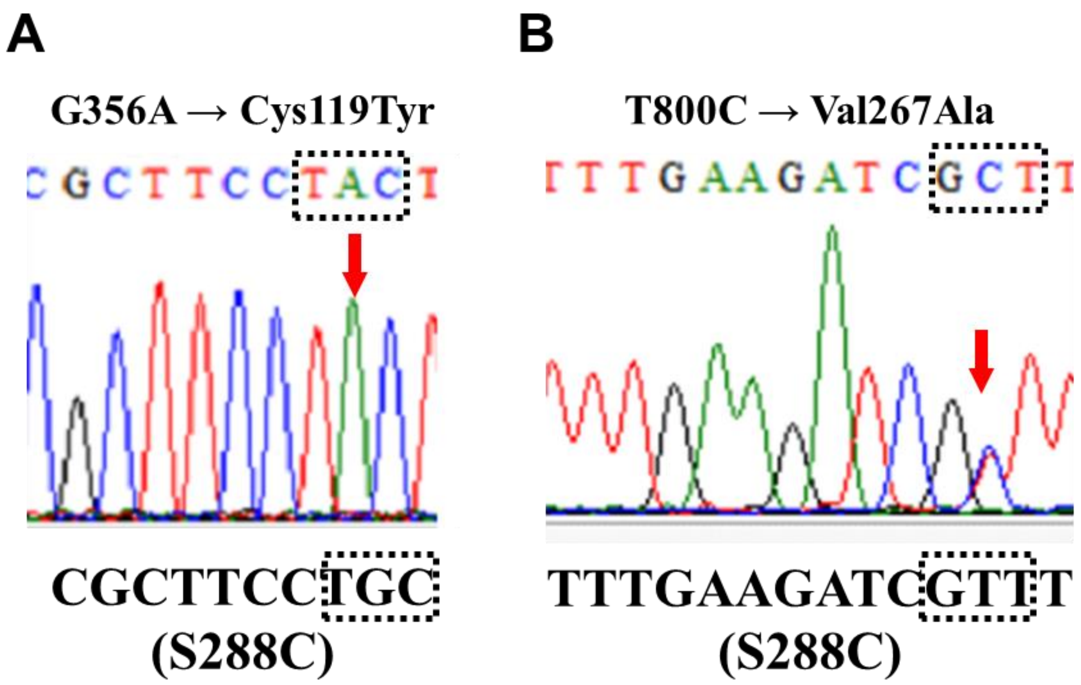

FIG S1 The DNA sequence of the *ARG5,6* gene in strains (A)A902-6 and (B) A902-8-3. The nucleotide sequences and corresponding amino acid residues of strains A902-6 and A902-8-3 are shown. The red arrows indicate the mutation of two strains compared with strain S288C. The sequence in A902-6 was A at position 356, leading one amino acid change of Cys to Tyr at position 119. The sequence in A902-8-3 was mixture of T and C at position 800, leading amino acid replacement of Val to Ala at position 267.

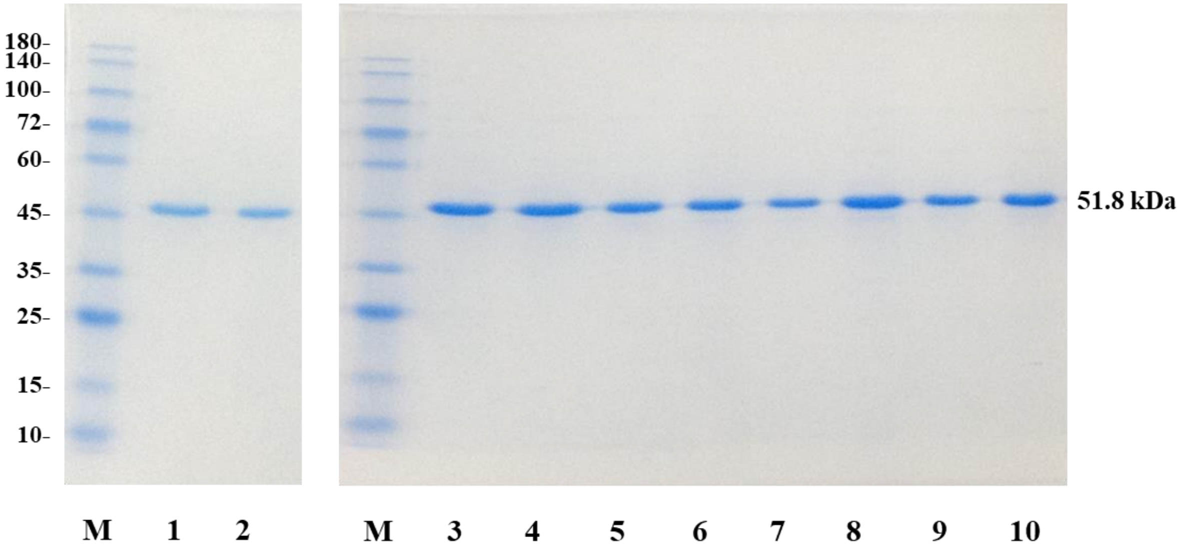

FIG S2 SDS-polyacylamide gel electrophoresis of the recombinant NAGKs (the wild-type and variants) purified from *E. coli* BL21 (DE3) cells. Lane M: Molecular mass standards (kDa), Lane 1: V267A, Lane 2: C119Y, Lane 3: T340A, Lane 4: T340L, Lane 5; T340R, Lane 6: T340E, Lane 7: T340N, Lane 8: T340S, Lane 9: WT

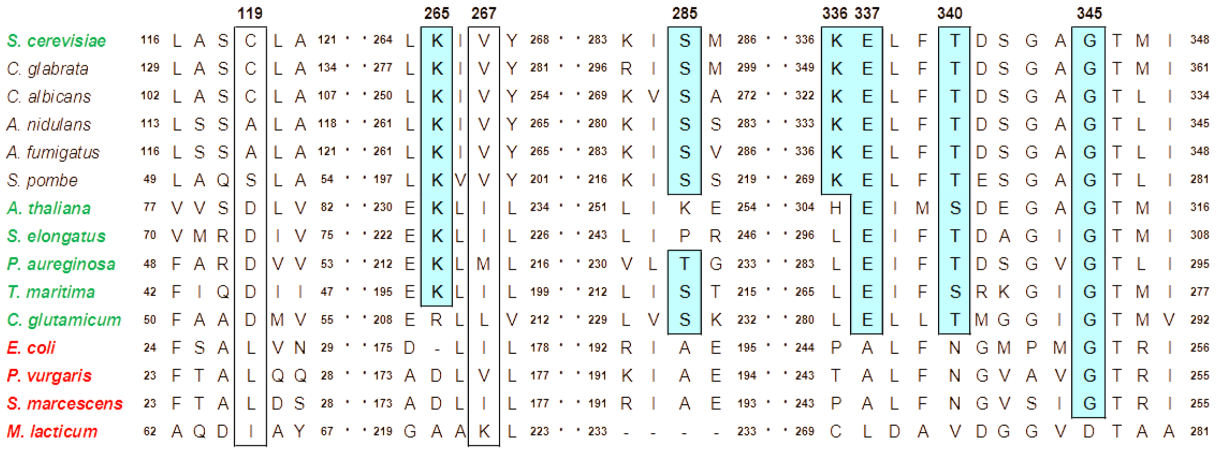

FIG S3 Multiple alignment of NAGKs from various organisms by ClustalW. Numbering of residues is in the *S. cerevisiae* NAGK. Among residues that interact with Arg, conserved residues were highlighted in cyan boxes. Microorganisms with green or red letter indicate organisms with Arg-sensitive or Arg-insensitive, respectively.

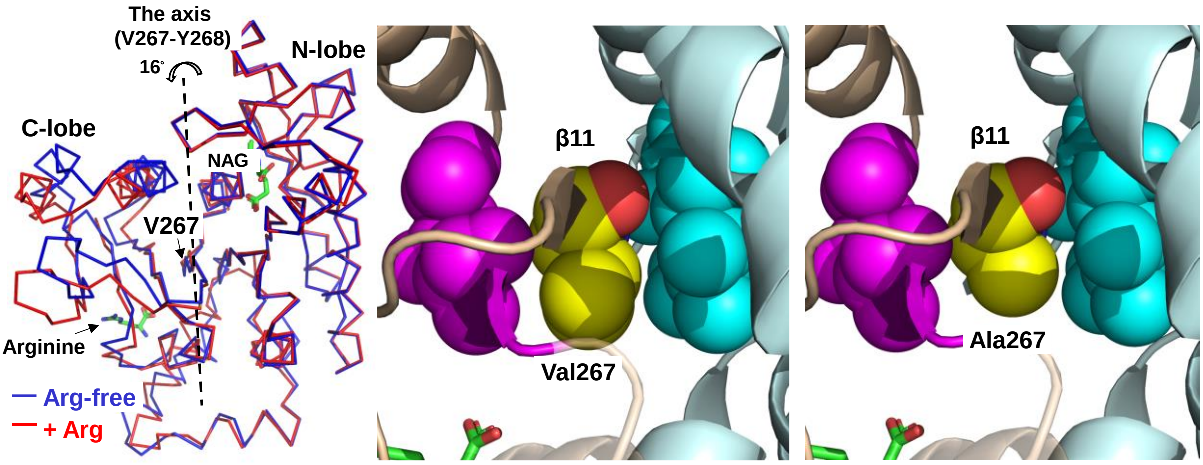

FIG S4 The structure of *S. cerevisiae* NAGK containing Arg. (A) The superimposition of the Arg-containing subunit (red) and of the Arg-free subunit (blue) of the same tetramer (subunits C and A (PDB ID code: 3ZZH), respectively) adapted from reference 26. The C-lobe of Arg-containing subunit rotates 16˚ around the drawn axis (black broken line) including the β11-sheet (V267-Y268 residues) compared with that of the Arg-free subunit. An inhibitor Arg and a substrate NAG are shown by the stick model (B and C) The enlarged structure located near the residue at position 267. Overall structure is shown by the carton model. Val326 and Ala327, Ala100, Val101, and Ile102 are represented as the sphere model in magenta and cyan, respectively. Val267 (B) and Ala267 (C) are also shown by the sphere model in yellow.
